# Draft Genome Sequences of 17 Isolates of the Plant Pathogenic Bacterium *Dickeya*

**DOI:** 10.1128/genomeA.00978-13

**Published:** 2013-11-21

**Authors:** Leighton Pritchard, Sonia Humphris, Gerry S. Saddler, John G. Elphinstone, Minna Pirhonen, Ian K. Toth

**Affiliations:** Information and Computational Sciences (ICS), James Hutton Institute, Invergowrie, Dundee, Scotland, United Kingdoma; Cellular and Molecular Sciences (CMS), James Hutton Institute, Invergowrie, Dundee, Scotland, United Kingdomb; Science and Advice for Scottish Agriculture (SASA), Edinburgh, United Kingdomc; Food and Environment Research Agency, Sand Hutton, York, United Kingdomd; Department of Agricultural Sciences, University of Helsinki, Helsinki, Finlande

## Abstract

*Dickeya* (formerly *Erwinia chrysanthemi*) species cause diseases on a wide range of crops and ornamental plants worldwide. Here we present the draft sequences of 17 *Dickeya* isolates spanning four *Dickeya* species, including five isolates that are currently unassigned to a species.

## GENOME ANNOUNCEMENT

Species of the *Dickeya* genus (previously *Erwinia chrysanthemi*) of phytopathogenic bacteria cause disease in a wide range of plant species, including crops, worldwide (1). The *Dickeya* genus is currently described as having six species: *dianthicola*, *dadantii*, *zeae*, *chrysanthemi*, and *paradisiaca* (2, 3) and the newly described *D. solani* (4). Draft genome sequences of eight *D. dianthicola* and *D. solani* isolates were recently described (5), and four complete sequences of *Dickeya* strains, *D. paradisiaca* strain Ech703 (accession no. NC_012880), *D. zeae* strain Ech586 (accession no. NC_013592), *D. chrysanthemi* strain Ech1591 (accession no. NC_012912), and *D. dadantii* strain Ech3937 (accession no. NC_014500), along with a draft *D. zeae* strain sequence, have been deposited in GenBank.

We announce draft genome sequences of 17 isolates of *Dickeya*, including isolates of *D. dadantii*, *D. chrysanthemi*, *D. zeae*, and *D. paradisiaca* and five isolates currently unassigned to a species ( Table 1). The 12 isolates assigned to species are *D. dadantii* type strain NCPPB 898, isolated from pelargonium (Comoro Islands); former *D. dadantii* subspecies *dieffenbachiae* type strain NCPPB 2976, isolated from dieffenbachia (United States); *D. dadantii* NCPPB 3537, isolated from potato (Peru); *D. chrysanthemi* type strain NCPPB 402, isolated from chrysanthemum (United States); *D. chrysanthemi* biovar parthenii NCPPB 516, from *Parthenium argentatum* (Denmark); *D. chrysanthemi* NCPPB 3533, isolated from potato (United States); *D. zeae* CSL RW192, isolated from river water (England); *D. zeae* MK19, isolated from river water (Scotland); the *D. zeae* phylotype II type strain NCPPB 2538, isolated from maize (United States); *D. zeae* NCPPB 3531 and NCPPB 3532, both isolated from potato (Australia); and *D. paradisiaca* type strain NCPPB 2511, isolated from plantain (Colombia). The five isolates with unassigned species are CSL RW240/1 from river water (England), DW0440 from river water (Finland), MK7 from river water (Scotland), NCPPB 569 from sugarcane (Australia), and NCPPB 3274 from *Aglaonema* (St. Lucia).

**TABLE 1:** Statistics for the 17 draft *Dickeya* genome sequences

Species	Strain	Accession no.	No. of contigs	No. of assembled bases	N_50_	No. of predicted coding sequences
***D. chrysanthemi***	NCPPB 402^T^	AOOA00000000	12	4,797,070	2,467,266	4,447
***D. chrysanthemi***	NCPPB 516	AOOC00000000	35	4,614,776	443,362	4,444
***D. chrysanthemi***	NCPPB 3533	AOOJ00000000	91	4,723,912	102,359	4,467
***D. dadantii***	NCPPB 898^T^	AOOE00000000	52	4,933,637	191,282	4,591
***D. dadantii***	NCPPB 2976^T^	AOOG00000000	84	4,810,532	114,781	4,552
***D. dadantii***	NCPPB 3537	AOOL00000000	47	4,805,222	222,170	4,430
***D. zeae***	NCPPB 2538^T^	AOOF00000000	46	4,559,915	237,408	4,225
***D. zeae***	NCPPB 3531	AOOI00000000	29	4,623,158	385,197	4,256
***D. zeae***	NCPPB 3532	AONW00000000	19	4,555,162	330,312	4,261
***D. zeae***	CSL RW192	AONY00000000	56	4,696,643	240,868	4,402
***D. zeae***	MK19	AOOR00000000	35	4,669,100	417,168	4,346
***D. paradisiaca***	NCPPB 2511^T^	AONV00000000	43	4,627,470	160,099	4,376
**Unassigned**	NCPPB 569	AOOD00000000	66	4,215,441	142,291	4,060
**Unassigned**	NCPPB 3274	AOOH00000000	62	5,110,316	166,211	4,729
**Unassigned**	CSL RW240/1	AONZ00000000	82	4,380,918	159,782	4,107
**Unassigned**	DW0440	AOON00000000	234	4,330,262	68,778	4,132
**Unassigned**	MK7	AOOO00000000	66	4,926,896	190,720	4,513

All strains were sequenced using 454 pyrosequencing (Roche, Branford, CT). Isolates NCPPB 402^T^ and NCPPB 2511^T^ were sequenced using paired-end libraries, and the remaining isolates were sequenced using single-end libraries. Sequences of nine strains (CSL RW192, CSLRW 240/1, DW0440, MK7, MK19, NCPPB 402^T^, NCPPB 2538^T^, NCPPB 2976^T^, and NCPPB 3531) were assembled *de novo* using 454 Life Sciences NEWBLER v2.5.3; meta-assemblies of NEWBLER *de novo* and reference-guided assemblies to the above-named sequences from GenBank were used for NCPPB 516 and NCPPB 3533 (to accession no. NC_012912); NCPPB 2511^T^ (to accession no. NC_012880); NCPPB 569, NCPPB 898^T^, NCPPB 3274, and NCPPB 3537 (to accession no. NC_014500); and NCPPB 3532 (to accession no. NC_013592).

Coding sequences for each isolate were annotated using Prodigal and RAST gene callers and BLAST using query sequences known to be missed by those packages. tRNAScan-SE was used to identify tRNA sequences. A detailed comparative genomic analysis of these and previously published draft *Dickeya* sequences (5) will follow in a future publication.

### Nucleotide sequence accession numbers.

The draft sequences of these *Dickeya* strains are available in GenBank under the following accession numbers: AONV00000000 (NCPPB 2511^T^), AONW00000000 (NCPPB 3532), AONY00000000 (CSL RW192), AONZ00000000 (CSL RW240), AOOA00000000 (NCPPB 402^T^), AOOC00000000 (NCPPB 516), AOOD00000000 (NCPPB 569), AOOE00000000 (NCPPB 898^T^), AOOF00000000 (NCPPB 2538^T^), AOOG00000000 (NCPPB 2976^T^), AOOH00000000 (NCPPB 3274), AOOI00000000 (NCPPB 3531), AOOJ00000000 (NCPPB 3533), AOOL00000000 (NCPPB 3537), AOON00000000 (DW0440), AOOO00000000 (MK7), and AOOR00000000 (MK19).
